# An Evaluation of White Matter Intensities in Patients with Pediatric Migraine

**DOI:** 10.3390/medicina61020186

**Published:** 2025-01-22

**Authors:** Burak Gülcen, Hilal Aydın, Erdoğan Bülbül, Bahar Yanik

**Affiliations:** 1 Department of Anatomy, Faculty of Medicine, Balıkesir University, 10145 Balıkesir, Turkey; 2 Department of Pediatric Neurology, Faculty of Medicine, Balıkesir University, 10145 Balıkesir, Turkey; drhilalaydin@gmail.com; 3 Department of Radiology, Faculty of Medicine, Balıkesir University, 10145 Balıkesir, Turkey; drerdoganbulbul@yahoo.com (E.B.); bhrynk@gmail.com (B.Y.)

**Keywords:** migraine, magnetic resonance imaging, white matter hyperintensities, pediatrics

## Abstract

*Background and Objectives*: This study aims to assess white matter hyperintensities (WMHs) in pediatric migraine patients and to elucidate the pathophysiology of the disease. *Materials and Methods*: A total of 30 patients diagnosed with migraine and 28 healthy individuals undergoing magnetic resonance imaging (MRI) scans for various reasons in our hospital between September 2019 and April 2023 were included in the study. We evaluated the presence, number, locations, and volumetric measurement of WMHs and the relationship between hyperintensity and attack profiles in migraine patients. *Results*: WMHs were observed at MRI in 18 (60%) of the 30 migraine patients and in 8 (28.6%) of the 28 controls. One hyperintense lesion was detected in nine members of the patient group, two in six patients, five in one patient, eight in one patient and nine in one. One hyperintense lesion was detected in eight healthy control group members. WMHs were significantly more common in the migraine patients than in the control group (*p* = 0.016). There was no significant relationship between hyperintensity in migraine patients and attack duration or frequency. Analysis also revealed no significant difference in terms of hyperintensity volumes (mm^3^) between the study group (19.73 ± 24.26) and the control group (5.62 ± 1.83). *Conclusions*: This study set out to show that migraine exerts neurological effects that are not solely limited to pain by emphasizing the pronounced differences observed on the brain MRIs of migraine patients. These findings may help us achieve a better understanding of the effects of migraine on cerebral structures and functions and identify therapeutic strategies in the future.

## 1. Introduction

Headache is one of the most frequently encountered complaints in the pediatric population [1]. Reported prevalence rates during childhood range from 25% to 93% [2,3,4,5].

Migraine is the most frequent cause of childhood headaches, affects approximately 15% of the community, and is characterized by recurrent attacks, albeit with a generally good course [6,7]. It is less common in children than in adults [8]. A systematic review of the literature reported an estimated migraine prevalence in children and adolescents of 7.7%. The prevalence of migraine is equal between the sexes until puberty [9].

Migraine is characterized by headache with a unilateral location, pulsating quality, moderate to severe intensity, a duration of 4–72 h, and aggravation by routine physical activity, as well as being associated with nausea, emesis, and/or photophobia and phonophobia [10]. Migraine sometimes directly follows unilateral, fully reversible neurological symptoms known as aura, which affect approximately one-third of migraine patients, last 5–60 min, and are most commonly visual or sensory in character [10,11].

Migraine in children is characterized by being shorter in duration than in adults and by frequently exhibiting a bilateral course [10].

The condition exhibits clinical features that can develop with age and that adversely impact education, socialization, and family life by significantly affecting the child’s quality of life [12]. Studies show that migraine is not limited to pain but can also lead to gradual changes in brain tissue [6].

Magnetic resonance imaging (MRI) of the brain is frequently performed as part of the workup for pediatric headache with the primary goal of ruling out causes of secondary headache such as intracranial masses or vascular anomalies. Most brain MRIs performed for the evaluation of headache in the pediatric population are normal [13]. However, incidental findings are frequently discovered, being reported in approximately one-fifth of studies involving generally healthy pediatric patients [14,15,16].

White matter hyperintensities (WMHs) are one of the most frequently encountered incidental findings on pediatric brain MRIs. White matter lesions (WMLs) are seen within various pediatric populations, including healthy controls, although their relationship with migraine has not been well defined [17].

WMHs, regarded as vascular in origin, are subclinical brain injuries representing damage to arterioles, capillary vessels, and venules [18].

A previous meta-analysis reported an increase in white matter microstructural abnormalities in patients with migraine [7]. In the pathophysiology of migraine, increased white matter is associated with cortical hyperexcitability, habituation deficiency, and sensitization responses, serving as an indicator of abnormal plasticity and neurological alterations. This increase is considered a critical biological marker linked to the chronic progression of migraine and changes in pain perception, underpinning the subclinical brain damage observed in patients. The investigation of habituation and sensitization responses represents a promising approach to understanding the effects and mechanisms of these changes in white matter within the context of migraine [19,20].

Various imaging techniques are applied in order to investigate the pathophysiology of migraine. MRI is particularly widely employed in such research. It is used to evaluate cerebral white matter intensities and has been described in such neurological lesions as migraine, ischemia, and multiple sclerosis [21].

The primary aim of this study was to evaluate the presence and extent of WMHs in patients with pediatric migraine and to assess their relationship with the pathophysiology of the disease. The secondary aim was to explore the potential association between microstructural changes in white matter and clinical parameters such as migraine frequency and severity. We assessed the relationship between WMH and factors such as migraine frequency, duration, severity, and gender.

## 2. Materials and Methods

A total of 30 patients diagnosed with migraine at the Balıkesir University Medical Faculty pediatric neurology clinic, Balıkesir, Türkiye, based on International Headache Society criteria (ICHD) (3rd Edition) between 1 September 2019 and 1 April 2023 and 28 healthy individuals were included in the study.

Individuals presenting with symptoms such as syncope, dizziness, nausea, tinnitus, and vertigo who underwent MRI examinations and were not using any medication were included in the control group.

All participants of both sexes were aged 1–18 years. Since the migraine patients attended follow-up visits at varying intervals, some were evaluated during periods of active pain, while others were assessed on pain-free days. No menstrual cycle data were obtained from either group.

Patients who underwent MRI in another hospital, with chronic diseases, with histories of drug use or other pain syndromes, using painkillers for more than eight days a month, who used illegal drugs, with psychiatric or other significant systemic disorders, with any mass or dysplasia observed at MRI, or with missing file data were excluded from the study.

The imaging was performed using a 1.5 Tesla MRI device (Philips Ingenia, Best, The Netherlands). The scanning protocol consisted of three-dimensional fluid-attenuated inversion recovery (FLAIR) images. The imaging parameters were repetition time (TR): 4800 ms, echo time (TE): 315 ms, inversion time (IT): 1660 ms, slice thickness: 1.04 mm, matrix 216/218, and field of view (FOV): 250 × 250 × 189 mm. No intravenous contrast material was employed under any conditions.

Approval for the study was obtained from the Balıkesir University Medical Faculty clinical research ethical committee (decision no. 2023/67—date 10 May 2023).

All MRI scans were evaluated retrospectively from 3D FLAIR images by a specialist radiologist and anatomist blinded to the patients’ clinical details. The presence, number, and locations of WMHs were recorded. Volumetric measurement of hyperintensities was conducted by two users on 3D Slicer software (3D Slicer software 10.4.2, http://www.slicer.org (accessed on 16 May 2019)). The region of interest was adjusted so as not to exceed the anatomical margins of the hyperintensity. Volume calculation was carried out once each section containing the relevant hyperintensity had been marked. Interobserver variability was set to less than 5%.

### Statistical Analysis

Demographic and clinical data were retrieved from the medical records. Data analysis was carried out on SPSS software v.22.0 (SPSS Inc., Chicago, IL, USA). Summary statistics included mean, standard deviation (SD), median, maximum, and minimum values for continuous variables and percentage and frequency values for categorical variables. The chi-square and Mann–Whitney U tests were applied for statistical comparisons. *p* values < 0.05 were regarded as statistically significant. Linear regression analysis was performed to evaluate the relationship between radiological and clinical findings.

## 3. Results

The control group consisted of 16 girls (57.1%) and 12 boys (42.9%), with a mean age of 10.68 ± 4.46 years. The migraine patient group was made up of 21 girls (70%) and 9 boys (30%), with a mean age of 13.20 ± 3.60 years (Table 1). Only 2 (6.7%) of the migraine patients were receiving pharmacotherapy, the other 28 (29.3%) being followed-up without treatment. The majority of patients experienced 2–6 attacks a week (n = 20, 66.7%), with the attack duration being usually four hours or less (n = 25, 83.3%) (Table 1).

Individuals with migraine were enrolled as the patient group, and the healthy cases as the control group.

**Figure 1:**
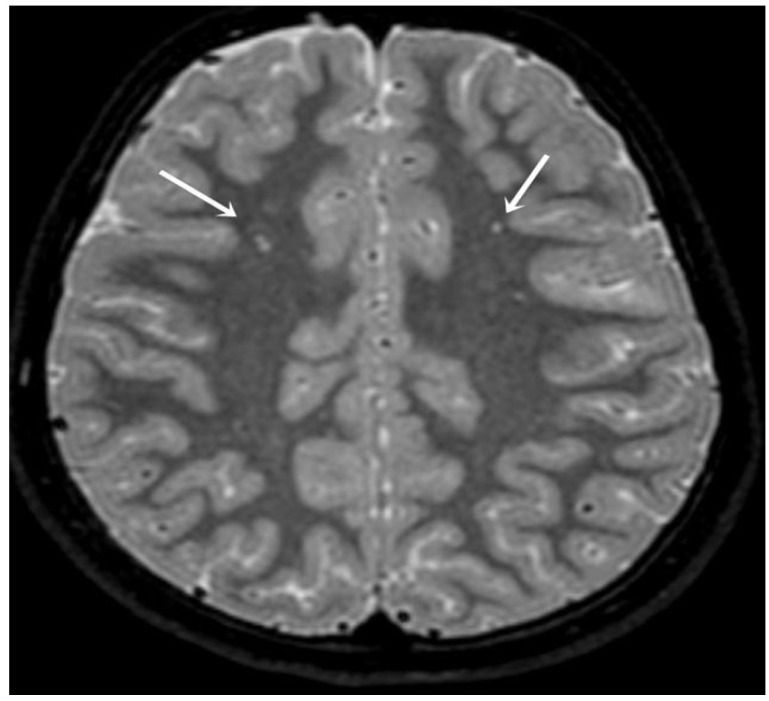
Bilateral subcortical hyperintensities (white arrows) on an axial plane FLAIR image from a 10-year-old girl exposed to frequent migraine attacks.

WMHs were observed at MRI in 18 (60%) of the 30 migraine patients and in eight (28.6%) of the 28 controls. One hyperintense lesion was detected in nine members of the patient group, two in six patients, five in one patient, eight in one, and nine in one. An example of bilateral hyperintensities observed on an axial plane FLAIR image is shown in Figure 1.

One hyperintense lesion was detected in eight healthy control group members. Comparison showed that WMHs were statistically significantly more common at MRI in the migraine patients than in the control group (*p* = 0.016). The sites of the hyperintensities in the two groups are shown in Table 2.

No significant difference was observed in hyperintensity volumes between the study group (19.73 ± 24.26 mm^3^) and the control group (5.62 ± 1.83 mm^3^) (*p* = 0.113) (Table 3).

Linear regression analysis revealed no significant relationship between hyperintensity in the migraine patients and attack duration (R = 0.79, R^2^ = 0.06, *p* = 0.678) or attack frequency (R = 0.24, R^2^ = 0.01, *p* = 0.901).

## 4. Discussion

This study examined the cerebral MRI findings of migraine patients and healthy controls. Previous studies have reported WMHs being observed incidentally in a healthy group, with higher rates of being reported on the cranial MRIs of patients with migraine compared to non-migraine patients [22,23]. Fisch et al. reported headache in five (31%) out of 16 children in whom WMHs were detected at MRI [24]. Another study examining the relationship between migraine and WMHs in children with headache determined such lesions in approximately 10% of migraine patients. The low incidence in that study was attributed to the shorter attack duration in pediatric patients [25]. In the present study, the incidence of hyperintensity in white matter in children was significantly higher in the patient group than in the healthy controls (*p* = 0.016). This figure was lower than that reported elsewhere among adult migraine patients. Nonetheless, white matter abnormalities were detected at MRI in 18 (60%) of the 30 members of the patient group in the present study. Relatively few studies have investigated the volume measurements of WMHs in adult migraine patients, and such research among pediatric migraine patients is exceedingly rare. From that perspective, the present WMH volume study among pediatric migraine patients conducted in association with attack durations and frequencies is valuable as a significant contribution to the current literature. No statistically significant difference in WMH volumes was observed in this research between the two groups (*p* = 0.113).

Another study involving cerebral MRI screening of pediatric migraine patients observed no white matter hyperintense lesions in a healthy control group but detected such lesions in 2 (13.3%) out of 15 migraine patients. Several small punctate T2 hyperintense lesions were detected in the deep and subcortical white matter in two pediatric patients with migraine without aura. Those authors observed abnormalities in the white matter tracts in children with migraine and suggested that these changes were related to the duration of the disease and the attack frequency. Based on those findings, they suggested that migraine is not only a painful condition, but also one that gradually affects the brain [6]. In the present study, WMHs were encountered at MRI in 18 (60%) of the 30 migraine patients. Such hyperintensities were statistically significantly more common in the migraine patients than in the control group (*p* = 0.016). Two other studies suggested that migraine may cause subclinical brain lesions and infratentorial hyperintensities [26,27]. Other studies have determined a significantly higher risk of WMHs in women, independently of the migraine subtype and cardiovascular risk factors [26,28]. There are also studies showing WMHs in different localizations in patients with migraine with aura. In their study from 2010, Rossato et al. reported that 19% of WMHs were sited in the periventricular region and 47% in deep white matter, and that 86% of the deep WMHs were located in the frontal region [29]. These findings indicate a neurological effect potential of migraine that is not solely limited to headache. Similarly, in the present study, WMHs were encountered in 8 (28.6%) of the 28 controls, but in 18 (60%) of the 30-member patient group.

Kruit et al. determined hyperintensities in the infratentorial region in 4.4% of migraine patients and 0.7% of a control group. Additionally, they reported that subclinical infarcts were more common in individuals with migraine, and that these lesions were greater in the posterior circulation region compared to those of the control group [27]. Consistent with the previous literature, WMHs were statistically significantly more common at MRI in the pediatric migraine patients compared to the healthy control group in the present research (*p* = 0.016). Additionally, and again in agreement with the literature, hyperintensities were most common in the frontal region (35.4%).

While this study is consistent with several findings suggesting that WMHs may be associated with migraine, it is important to acknowledge that other studies have yielded different results, indicating that the relationship between WMHs and migraine may not be completely straightforward. For example, a recent study by Ackley et al. found that T2 hyperintense WMLs are common in the pediatric population and do not appear more frequently in pediatric patients with migraine or other primary headache disorders. Those authors concluded that such lesions are likely incidental and unrelated to headache history. This is in contrast to our findings, in which we observed an association between WMHs and migraine patients [17]. Moreover, a population-based imaging study by Honningsvåg et al. found that individuals with tension-type headaches exhibited more WMHs than those without headaches. However, migraine and unspecified headache types had no significant effect on the prevalence of WMHs. This further highlights the variability in the relationship between headache disorders and such hyperintensities [30].

These inconsistent findings suggest that there may be a complex, multifactorial relationship between WMHs and headache disorders, including migraine. The results of the present research, which show an association between WMHs and migraine, may have been affected by factors not examined in studies reporting differing outcomes, such as patient demographics, the specific type and frequency of headaches, and potential confounding variables such as comorbidities or other neurological conditions. Further research, particularly involving larger and more diverse patient populations, is now needed to clarify the nature of this relationship and to explore the mechanisms underlying the presence of WMHs in migraine patients.

It has been suggested that WMHs may represent ischemic lesions associated with recurrent migraine attacks [31]. Several authors have reported that the frequency and duration of attacks can be indicative of brain damage [32]. However, linear regression analysis in the present study revealed no significant relationship between the hyperintensity seen in migraine patients and the duration of attacks (R = 0.79, R^2^ = 0.06, *p* = 0.678) or their frequency (R = 0.24, R^2^ = 0.01, *p* = 0.901). Analysis also revealed no significant difference in terms of hyperintensity volumes (mm^3^) between the migraine group (19.73 ± 24.26) and the control group (5.62 ± 1.83) (*p* = 0.113). Examining the potential mechanisms underlying the increase in white matter lesions in migraine patients may help deepen our understanding of the disease’s pathophysiology and help shape future approaches to treatment. Changes in brain structure due to migraine or chronic pain may lead to impairments in executive functions, particularly in cognitive processes such as working memory, inhibition, and cognitive flexibility.

In recent years, therapeutic methods such as cognitive training and dual-task protocols have emerged as innovative approaches for addressing these clinical and neurophysiological manifestations in migraine patients. Specifically, one study found that a dual-task protocol applied to episodic migraine patients was more effective than active exercise or cognitive training alone, yielding positive effects on both peripheral and central neurophysiological outcomes [33]. Another study indicated that migraine patients experience impairments in executive functions, which are associated with disability during migraine attacks [34]. Researchers have shown that patients with chronic pain also suffer from impairments in executive functions, which may in turn affect pain perception and coping strategies [35]. These findings suggest that cognitive training and dual-task protocols may serve as potential therapeutic options for improving executive functions and supporting pain management in patients with migraine and chronic pain. However, further research is needed to assess the effectiveness of these approaches.

Gender differences in migraine prevalence and severity are well-documented, with females often experiencing more frequent and severe attacks [36]. This disparity is often attributed to hormonal fluctuations [37,38]. In the present study, girls were more numerous in the migraine patient group. While understanding the role of gender and hormonal effects in migraine is crucial for developing targeted treatment strategies, this study did not specifically investigate their impact on white matter hyperintensities (WMH) or brain architecture. Further research could examine the potential effects of gender and hormonal fluctuations on WMH and cortical/subcortical changes in migraine patients.

The principal limitations of this study include the small sizes of the patient and control groups, its retrospective nature, and the difference in mean age between the two groups. While these limitations may have influenced the results, further multicenter studies with larger sample sizes and prospective designs are needed to confirm these findings and explore additional factors, such as menarche, that might contribute to a more comprehensive understanding of the relationship between migraine and brain changes.

## 5. Conclusions

Migraine emerges in association with a number of causes, the etiopathogenesis being highly complex and still unexplained. This study attempted to show that migraine exerts neurological effects that are not solely limited to pain by emphasizing the pronounced differences observed on the brain MRIs of migraine patients. WMHs were encountered in 18 (60%) of the 30 migraine patients and in 8 (28.6%) of the 28 controls. All the control group brain MRIs were normal, and no dysplasia, mass, or hyperintense lesions were detected. This finding was statistically significant (*p* = 0.016). Analysis of hyperintensity volumes (mm 3) revealed no significant difference between the patient and control groups. Additionally, no statistically significant association was determined between the hyperintensity observed in the migraine patients and the attack duration or frequency. These findings may help us achieve a better understanding of the effects of migraine on cerebral structures and functions and identify therapeutic strategies in the future. We think that this research is particularly valuable from that perspective due to the paucity of similar studies on the subject. However, the limitations of this study should be borne in mind, and we await the results of future research involving larger patient groups with interest.

## Figures and Tables

**Table 1:** The groups’ characteristics and hyperintensity frequencies.

	Control Group (n = 28)	Migraine Group (n = 30)	*p*
Gender n (%)			
Male	12 (57.1)	9 (30)	
Female	16 (42.9)	21 (70)	
Age (min–max)	10.68 ± 4.46 (3–17)	13.20 ± 3.60 (1–18)	
Drug use n (%)			
Yes		2 (6.7)	
No		28 (93.3)	
Attack duration n (%)			
<1 h		12 (40)	
1–4 h		13 (43.3)	
4–24 h		3 (10)	
>24 h		2 (6.7)	
Attack frequency n (%)			
Daily		6 (20)	
2–6/week		20 (66.7)	
1/week		4 (13.3)	
1/month		-	
MRI Hyperintensity n (%)			0.016
Yes	8 (28.6)	18 (60)	
No	20 (71.4)	12 (40)	

**Table 2:** Hyperintensity locations in the control and patient groups.

Control Group (n = 8)	n	%	Migraine Group (n = 18)	n	%
Right frontal subcortical	1	12.5	Right frontal subcortical	3	16
Right frontal	1	12.5	Right frontal	1	5.6
Right pericallosal	1	12.5	One right, one left parietal subcortical	1	5.6
Left anterior frontal subcortical	1	12.5	Two left frontal subcortical	1	5.6
Left frontal subcortical	1	12.5	Left basal ganglia level	1	5.6
Left parietal subcortical	1	12.5	Left frontal subcortical	1	5.6
Left pericallosal	2	25	Left occipital	1	5.6
			Left parietal	1	5.6
			Left temporal	1	5.6
			Four right, one left subcortical. Three of those on the right frontal, one centrum semiovale. Parietal on the left	1	5.6
			Two right pericallosal	1	5.6
			Two right, seven left bifrontal	1	5.6
			Two left frontal subcortical	1	5.6
			Two left frontal and parietal	1	5.6
			Two left corpus callosum	1	5.6
			Three right, five left bifrontal subcortical	1	5.6

**Table 3:** Hyperintensity volumes and distributions by groups.

	Control Group (n = 8)	Migraine Group (n = 18)	*p*
Gender n (%)			
Male	4 (50)	5 (27.8)	
Female	4 (50)	13 (72.2)	
Years (min–max)	11.38 ± 3.66 (5–16)	12.78 ± 4.05 (1–18)	
MR Hyperintensity Volume (mm^3^)			0.113
Min	3.36	3.02	
Max	8.39	95.67	
Mean SD	5.62 ± 1.83	19.73 ± 24.26	

## Data Availability

The data presented in this study are available on request from the corresponding author due to reasonable request.
